# Regional Homogeneity of Intrinsic Brain Activity in Happy and Unhappy Individuals

**DOI:** 10.1371/journal.pone.0085181

**Published:** 2014-01-15

**Authors:** Yangmei Luo, Xiting Huang, Zhen Yang, Baolin Li, Jie Liu, Dongtao Wei

**Affiliations:** 1 Key Laboratory of Cognition and Personality (SWU), Ministry of Education, Chongqing, China; 2 Faculty of Psychology, Southwest University, Chongqing, China; 3 Center for the Developing Brain, Child Mind Institute, New York, New York, United States of America; Institute of Psychology, Chinese Academy of Sciences, China

## Abstract

**Background:**

Why are some people happier than others? This question has intrigued many researchers. However, limited work has addressed this question within a neuroscientific framework.

**Methods:**

The present study investigated the neural correlates of trait happiness using the resting-state functional magnetic resonance imaging (rs-fMRI) approach. Specifically, regional homogeneity (ReHo) was examined on two groups of young adults: happy and unhappy individuals (N = 25 per group).

**Results:**

Decreased ReHo in unhappy relative to happy individuals was observed within prefrontal cortex, medial temporal lobe, superior temporal lobe, and retrosplenial cortex. In contrast, increased ReHo in unhappy relative to happy individuals was observed within the dorsolateral prefrontal cortex, middle cingulate gyrus, putamen, and thalamus. In addition, the ReHo within the left thalamus was negatively correlated with Chinese Happiness Inventory (CHI) score within the happy group.

**Limitations:**

As an exploratory study, we examined how general trait happiness is reflected in the regional homogeneity of intrinsic brain activity in a relatively small sample. Examining other types of happiness in a larger sample using a multitude of intrinsic brain activity indices are warranted for future work.

**Conclusions:**

The local synchronization of BOLD signal is altered in unhappy individuals. The regions implicated in this alteration partly overlapped with previously identified default mode network, emotional circuitry, and rewarding system, suggesting that these systems may be involved in happiness.

## Introduction

Although happiness is almost everyone's pursuit, the capacity to be happy varies widely across people. Why are some people happier than others? A large amount of correlational research addressed this question by examining the associations between happiness and a diverse range of factors, such as wealth, marriage, and life events [1]. Rather than focusing on the objective determinants of happiness, the construal theory focused on explaining the individual differences in happiness with hedonically related subjective psychological processes such as self-evaluation, self-reflection, self-regulation, social comparison, and person perception [2], [3]. This theory suggests that our alternative perspectives (either positive or negative) and our constructions of reality have different hedonic consequences and, as such, are associated with different levels of enduring happiness [2]. Within this framework, a wealth of behavioral studies have shown that happy and unhappy individuals have their own temperaments in hedonically related cognitive and motivational processes. Specifically, happy individuals, unlike their unhappy peers, appear to spend less time on self-reflection, are less sensitive to negative achievement feedback [3], experience positive emotion most of the time [4], have superior self-regulation abilities to maintain positive affective information in working memory [5], and have rich and more satisfying social relationships [4].

To date, limited work has investigated happiness within a neuroscientific framework [6]. Only a handful of studies have directly addressed the neural bases of trait happiness, which characterizes individuals' characteristic level of happiness during a particular period of time [7]. One resting-state electroencephalography (EEG) study revealed that individuals with greater activation in the left rather than the right superior prefrontal cortex were happier [8]. More recently, a structural magnetic resonance imaging (MRI) study demonstrated that eudaimonic happiness, which focuses on meaning and self-realization, was positively associated with gray matter volume in the right insular cortex [9]. Furthermore, a task-based functional MRI study focusing on emotional processes found that happier individuals showed greater amygdala responses to positive stimuli [10]. Thus, no resting-state fMRI research has been conducted to examine how trait happiness is reflected in the human brain.

Resting-state fMRI research has been blooming during the last couple of decades [11]–[13] since the seminal work by Biswal et al (1995) [14]. Not limited by tasks, this approach has been demonstrated as a powerful tool that can reliably characterize intrinsic brain activity [15]–[18]. In the present study, we used regional homogeneity (ReHo) [19], a widely used resting-state fMRI measure, to examine whether the local synchronization of spontaneous brain activities was associated with trait happiness. We were particularly interested in this measure for four reasons: (1) as a data-driven method, ReHo dose not require a priori hypothesis and is suitable for exploratory analysis; (2) the test-retest reliability of ReHo is well established. With an optimized acquisition and preprocessing pipeline, ReHo has been demonstrated as a highly reliable measure to map the regional activity of the human functional connectome [15]; (3) ReHo is associated with a variety of phenotypic variables, including relatively stable traits such as intelligence [20] and personality [21], suggesting that ReHo is able to capture the trait properties reflected in the intrinsic brain activity; and (4) ReHo has also been shown sensitivity to various neuropsychiatric disorders related to mood/emotional changes, such as depression [22], [23] and social anxiety disorders [24].

In the current study, we focused on obtaining preliminary knowledge on the neural signature of happiness by examining whether happy and unhappy individuals differed in ReHo. Building on the evidence that happy and unhappy individuals differed in self-evaluation, self-reflection, self-regulation, and person perception [2]–[5], we predict that the two groups will differ in ReHo within regions implicated in these functions.

## Materials and Methods

### Ethics statement

This study was approved by the Ethics Committee of the Southwest University. Written informed consent was obtained from all participants and they were informed that they can quit at any time during the experiment.

### Participants

**Figure 1:**
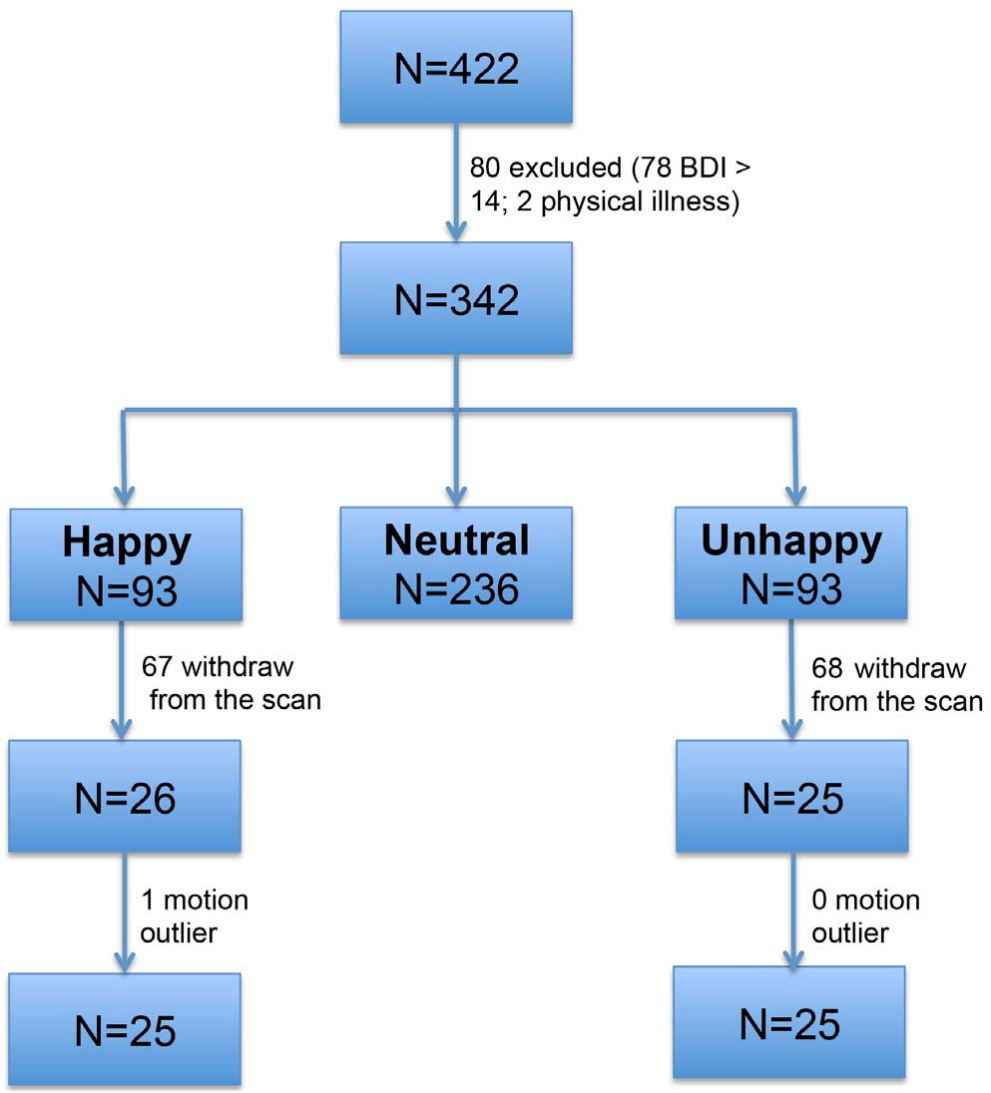
Participants flow chart.

A total of 422 undergraduate students were sampled at the Southwest University in China and assessed with the Subjective Happiness Scale (SHS) [25] and the Beck depression inventory (BDI) [26]. Seventy-eight participants were excluded from the current study due to mild to moderate depression (BDI scores >14) [27] and another 2 participants were excluded due to physical illness. The remaining 342 healthy participants were ranked according to the SHS scores. Given that 25 to 27 percent is most powerful in the extreme groups design [28], the upper and the lower 27% were selected as the potential happy and unhappy group. The middle 46% were discarded from the current study. We started with a relatively large sample to anticipate a high dropping rate of the scan. As we expected, a large number of participants in the selected two groups withdrew from the study due to either being unwilling or unavailable to participate the scan. We finally acquired imaging data on 51 participants (see Figure 1 for participants' number change at each step. happy group: N = 26, SHS = 6.50±0.29; unhappy group: N = 25, SHS = 3.96±0.45).

The four-item SHS has been frequently used to assess overall dispositional happiness and is appropriate for different ages, occupations and cultural groups [25]. SHS has demonstrated good internal consistency (Cronbach's α = 0.86) and high validity with Chinese Happiness Inventory (CHI, full version) [29] (*r* = 0.604, *p*<0.001).

### Data acquisition

The experiment was performed on a 3 Tesla Siemens Tim Trio system (Siemens, Erlangen, Germany). Functional images were acquired using a single-shot, gradient-recalled echo planar imaging sequence (TR = 2000 ms, TE = 30 ms, flip angle = 90°, 32 axial slices, FOV = 192 mm×192 mm, acquisition matrix = 64×64, slice thickness  = 3 mm, with 1 mm gap, voxel size  = 3 mm×3 mm×4 mm). For each participant, a total of 8 minutes of resting data were acquired. Participants were instructed to simply rest with their eyes closed, not to think of anything in particular, and not to fall asleep. To minimize head motion, participants' heads were restricted with foam cushions. For spatial normalization and localization, high-resolution T1-weighted anatomical images were also acquired in sagittal orientation using a 3D magnetization prepared rapid gradient-echo (MPRAGE) sequence (176 slices, TR = 1900 ms, TE = 2.53 ms, flip angle  = 9°, resolution  = 256×256, and voxel size  = 1 mm×1 mm×1 mm) on each participant.

Following the scan, participants' affective states were assessed with the Positive and Negative Affect Schedule (PANAS) [30]. To further confirm the effectiveness of grouping using SHS, participants were assessed with CHI within one week after the scan. One participant in each group failed to complete the CHI, leaving 24 participants with valid CHI data in each group.

### Data preprocessing

Data were preprocessed using DPARSF (Data Processing Assistant and Resting-State FMRI, version 2.2) [31] with the following steps: (1) removing the first ten volumes to account for the T1 equilibrium effect, leaving 230 volumes for final analysis; (2) slice timing correction; (3) motion correction by realigning images to the first volume then to the mean functional image; (4) segmenting T1 images into gray matter (GM), white matter (WM) and cerebrospinal fluid (CSF); (5) regressing out 27 nuisance covariates (including signals from WM, CSF, global signal, and Friston 24 motion parameters) to reduce the potential effects of physiological processes and motion. The Friston 24-parameter model (i.e., 6 head motion parameters, 6 head motion parameters one time point before, and the 12 corresponding squared items) [32] was used to regress out head motion effects based on recent work showing that higher-order models were more effective in removing head motion effects [33], [34]. The linear trends were also removed; (6) spatially normalizing the functional images to the Montreal Neurological Institute (MNI) space using the standard EPI template in SPM8 and resampling the images at a resolution of 3 mm×3 mm×3 mm; and (7) temporally band-pass filtering (0.01<f<0.08 Hz).

To further rule out the residual effect of motion on ReHo, volume-level mean framewise displacement (FD) was computed [35]. Participants with excessive motion (outside of the 3 inter-quartile range) relative to their group were excluded as outliers. One participant from the happy group was identified as an outlier and eliminated from the analyses. The final two groups did not differ in mean FD (*t* [48]  = 0.50, *p* = 0.42; happy: 0.13±0.06 mm; unhappy: 0.14±0.06 mm).

### Individual-level analysis

For each individual, a ReHo map was generated using REST (Resting state fMRI data analysis toolkit, version 1.8) [36]. Specifically, the Kendall's coefficient of concordance (KCC) of each voxel was calculated with its nearest neighbors (26 voxels) in a voxel-wise analysis. The formula for calculating the KCC value has been expounded in a previous study [19]. To reduce the influence of individual variations in the KCC value, standardization of ReHo maps were done by dividing the KCC of a given voxel by the averaged KCC of the whole brain. Then, the standardized ReHo maps were smoothed with a Gaussian kernel of 4 mm full-width at half-maximum to reduce noise.

### Group-level analysis

One-sample t-tests were first performed within each group to detect where the standardized KCC values were larger than the global mean KCC. The results were false discovery rate (FDR) corrected at *p*<0.05 [37].

To examine the group differences in ReHo, voxel-wised two-sample t-tests were performed on ReHo maps using the statistical program in the REST toolkit. Mean FD and gender were included as nuisance covariates to remove the residual effect of motion and gender effect at the group level. Following previous studies [38], [39], the t-map was masked by a grey matter map, which was obtained by segmenting the mean normalized high resolution T1-weighted images of all the participants to include only the areas falling in grey matter. Multiple comparisons were corrected using Monte Carlo simulation (The AlphaSim program in REST software). The Parameters were: single voxel *p* = 0.01, combined height threshold *p*<0.05 and a cluster size >486 mm^3^, 5000 simulations, FWHM  = 4 mm, cluster connection radius r = 5 mm).

### Brain-behavior relationships

To explore whether ReHo correlates with the level of happiness, Pearson's correlations between the mean ReHo extracted from regions showing significant group differences and the subjective happiness scores (SHS and CHI) were computed for happy and unhappy group, respectively.

## Results

### Demographical and neuropsychological results

The demographical and neuropsychological results were presented in Table 1. The happy and unhappy group did not differ in age and gender (*p*s>0.20) and as expected, significantly differed in subjective happiness measured with SHS and CHI (*p*<0.001).

**Table 1:** The Sample Characteristics.

	Happy group (*N* = 25)	Unhappy group (*N* = 25)	
Age (mean, *SD*)	20.16(1.34)	20.36(1.38)	*t*(48) = −0.52, *p* = 0.606
Gender (male/female)	6/19	8/17	*x^2^* (1) = 0.40, *p* = 0.754
SHS (mean, *SD*)	6.51(0.29)	3.96(0.45)	*t*(48) = 23.55, *p*<0.001
PA (mean, *SD*)	33.96(3.9)	29.84(5.21)	*t*(48) = 2.38, *p* = 0.021
NA (mean, *SD*)	18.00(3.74)	20.80(5.21)	*t*(48) = −2.23, *p* = 0.031
CHI (mean, *SD*)	2.621(0.33)(*N* = 24)	2.21(0.30)(*N* = 24)	*t*(46) = 4.42, *p*<0.001

*Note*: SHS =  Subjective Happiness Scale; PA =  Positive Affect (subscale of Positive and Negative Affect Schedule); NA =  Negative Affect (subscale of Positive and Negative Affect Schedule); CHI =  Chinese Happiness Inventory.

### Group analysis in ReHo

**Figure 2:**
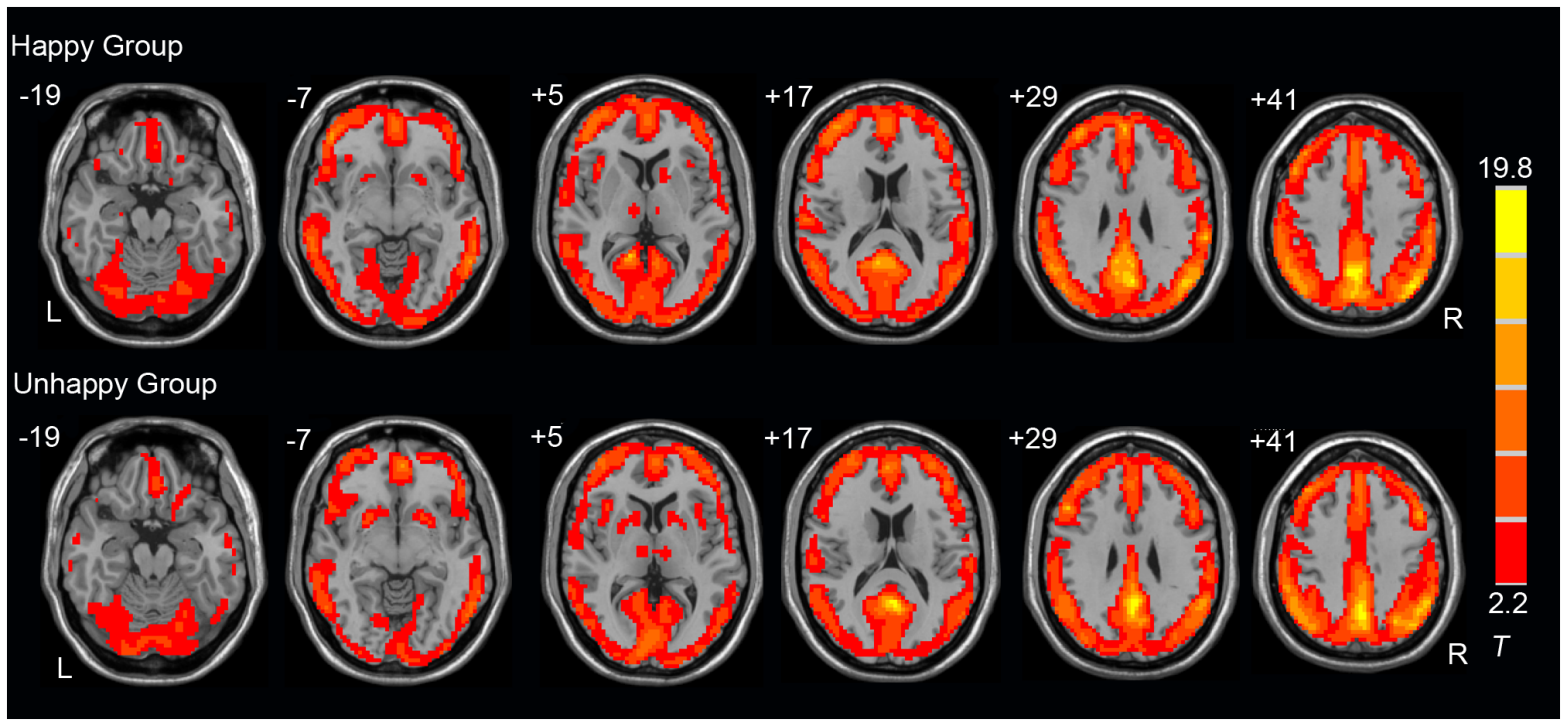
Mean ReHo map for each group. The t statistical maps for the Happy (top panel) and the Unhappy (bottom panel) group are presented (one-sample t-test, *p*<0.05, FDR corrected). Locations of the axial (Z) slices are given according to MNI space. L: left; R: right.

The mean ReHo maps for the happy and unhappy group are shown in Figure 2 (one-sample t-test; *p*<0.05, FDR corrected). The default-mode network (DMN), mainly including the medial prefrontal cortex (MPFC), posterior cingulate cortex (PCC), and bilateral parietal lobel, has higher regional homogeneity than other brain regions (Figure 2). This pattern is similar in the two groups and is consistent with previous studies [19], [40], [41].

**Figure 3:**
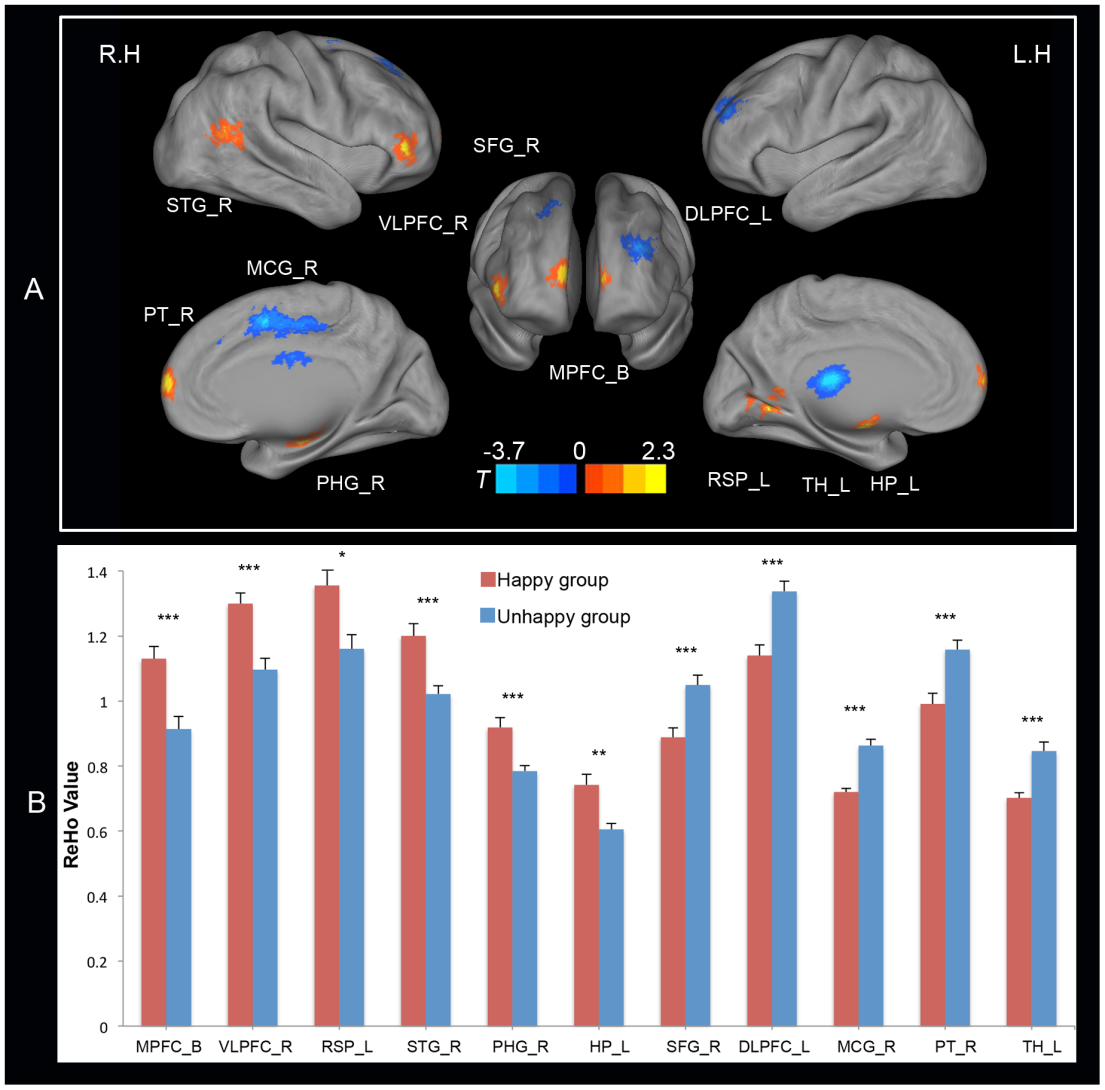
Brain regions exhibiting significant group effect and their mean ReHo scores. **Panel A** presents brain regions exhibiting significant group effect (two sample t-tests, thresholded at *p*<0.05, AlphaSim corrected). Statistical maps (lateral, medial and anterior views) were projected onto the PALS (population-average, landmark, and surface-based) atlas using CARET software [72]. The magnitude and direction of the t scores are represented by either warm (happy > unhappy) or cool (unhappy > happy) coloring. R.H. represents right hemisphere and L.H. represents left hemisphere. **Panel B** presents the mean ReHo scores for the Happy (red bars) and Unhappy (blue bars) group within the regions showing significant group effect. Error bars correspond to the standard error of mean. *: *p*<0.05, **: *p*<0.01, and ***: *p*<0.001. Abbreviations: MPFC_B =  bilateral medial prefrontal cortex; VLPFC_R  =  right ventrolateral prefrontal cortex; RSP_L  =  left retrosplenial cortex; STG_R  =  right superior temporal gyrus; PHG_R  =  right Parahippocampa Gyrus; HP_L  =  left hippocampus; SFG_R  =  right superior frontal gyrus; DLPFC_L  =  left dorsolateral prefrontal cortex; MCG_R  =  right middle cingulate gyrus; PT_R  =  right Putamen; TH_L  =  left Thalamus.

The two-sample t-tests results indicated that several regions were implicated in the significant group differences in ReHo (Figure 3 and Table 2). Specifically, compared with the happy individuals, the unhappy individuals exhibited significantly decreased ReHo in the bilateral MPFC, the right ventrolateral prefrontal cortex (VLPFC), the right superior temporal gyrus (STG), the left hippocampus (HP), the right parahippocampal gyrus (PHG), and the left posterior cingulate cortex/retrosplenial cortex (PCC/RSP). In contrast, increased ReHo was observed within the left dorsolateral prefrontal cortex (DLPFC), the right superior frontal gyrus (SFG), the right middle cingulate gyrus (MCG), the right putamen (PT), and the left thalamus (TH).

**Table 2:** Brain regions exhibiting significant group differences in ReHo.

Anatomical region	Side	BAs	MNI			Voxel Size (voxels)	Peak T-value
			x	y	z		
**Happy > Unhappy**							
Medial Prefrontal Cortex	B	10	−6	72	6	39	3.39
Ventrolateral Prefrontal Cortex	R	45	48	42	−3	20	4.33
Posterior Cingulate Cortex/Retrosplenial Cortex	L	18	−9	−57	3	19	3.25
Superior Temporal Gyrus	R	22	51	−57	12	22	3.55
Parahippocampa Gyrus	R		18	−12	−18	25	3.86
Hippocampus	L		−15	−9	−12	18	3.52
**Unhappy > Happy**							
Superior Frontal Gyrus	R	9	21	36	48	23	−3.53
Dorsolateral Prefrontal Cortex	L	46	−30	51	24	45	−4.47
Middle Cingulate Gyrus	R	6	15	0	51	120	−5.77
Putamen	R		27	9	−6	23	−3.4
Thalamus	L		−6	−21	15	41	−4.27

*Note*: Side refers to the hemisphere side (B: bilateral; R: right; and L: left). The Brodmann areas (BAs), the coordinates of peak t-value in Montreal Neurological Institute (MNI) space, the volume in voxels, and the peak t-value are specified for each region showing group differences in regional homogeneity (ReHo).

### Brain-behavior correlations

**Figure 4:**
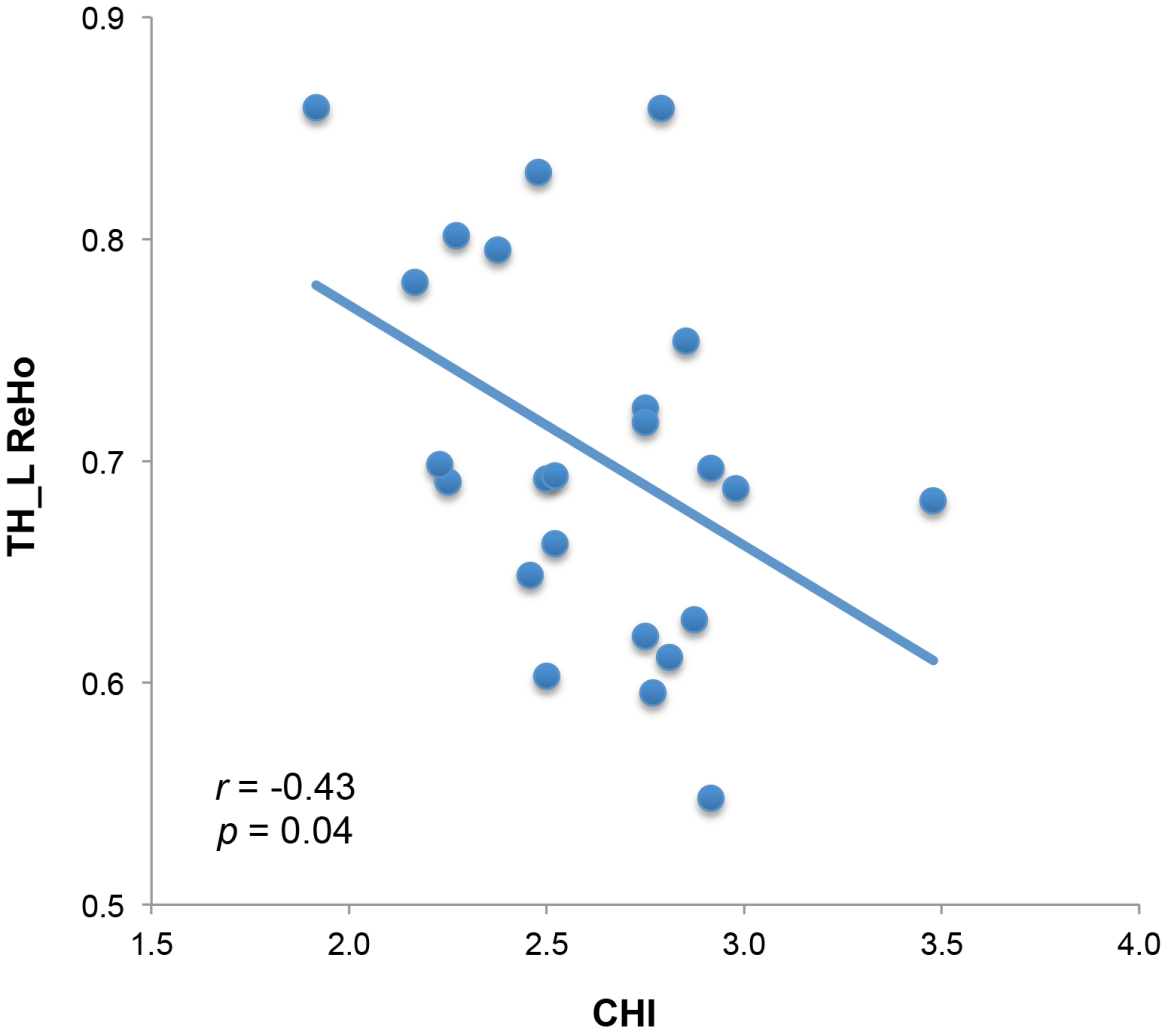
The mean ReHo within the left Thalamus (TH_L) is negatively correlated with the Chinese Happiness Inventory (CHI).

For the happy group, there was a significant negative correlation between the CHI and the mean ReHo within the left TH (Figure 4, *r* = −0.43, *p* = 0.04, uncorrected). No other correlations were significant (*p*s>0.05).

## Discussion

The present study used resting-state fMRI approach to examine whether local functional homogeneity was modulated by happiness. Significant group differences in ReHo were observed within distributed brain regions over prefrontal cortex, temporal lobe, limbic system and certain subcortical regions, suggesting multiple brain regions were involved in trait happiness. In addition, the ReHo within left thalamus was correlated with CHI in happy group, suggesting that ReHo could be useful for indexing the extent of happiness. Recently, extensive studies have reported altered ReHo in clinical populations with emotional disorders, such as depression [22], [23] and social anxiety disorders [24]. The novelty of the current study is that we observed alterations of ReHo in healthy individuals who experience more negative affect.

ReHo measures the local synchronization of a given voxel with its nearest neighbors based on the assumption that if a brain region is responsible for a specific function, the voxels within this region were more temporally homogeneous when involved in that function [19]. The ReHo results during a unilateral finger movement is partly consistent with electrophysiologic studies [19] and resting state ReHo is of functional relevance and can predict the behavioral performance of a stop signal task [42]. These results suggest that altered ReHo might reflect changes in the temporal feature of intrinsic neuronal activity.

Compared to happy individuals, unhappy individuals exhibited decreased ReHo within MPFC, VLPFC, medial temporal lobe (MTL), STG, and PCC/RSP cortex. These regions largely overlap the DMN, suggesting the involvement of DMN in happiness. This is supported by the reports that ReHo was altered within the DMN in depression [43], [44] and bipolar depression disorder [45]. DMN activity during rest is associated with mind-wandering or stimulus-independent thoughts [46] and a higher frequency of mind-wandering has been shown to be related to less happiness [47]. However, recent work demonstrated that it may not be mind-wandering per se that is responsible for psychological distress, but rather the general tendency to be less aware and attentive to the present-moment [48].

Happy and unhappy individuals have their own characteristic temperaments. Individuals with lower level of happiness spent more time ruminating on negative feelings, thoughts and shortcomings [2], [3], [47], have less satisfying social function [49], and envision future with less details [50]. These characteristics may be explained by the abnormalities in the DMN, which is involved in personal significance evaluation, self-relevant affective decision-makings, present mental states consideration, inference of other individuals' mental states, and envision future events [51]. The involvement of DMN in happiness also supports the construal theory which suggests self-reflection, self-evaluation, social comparison and personal perception are psychological processes that influence our constructions of reality and have hedonic consequences [2].

Converging evidence from lesion, neuroimaging and electrophysiological data supports the view that the prefrontal cortex (PFC) is a key component of the circuitry that implements both positive and negative affect [52]. In our study, we found that several important subdivisions of PFC are involved in trait happiness, such as MPFC and DLPFC. MPFC played an important role in hedonic evaluation of pleasure valence [6]. Compared to healthy participants, decreased ReHo was observed in depression [22], [53] and social anxiety disorder [24]. Furthermore, after a 6-week duloxetine therapy, ReHo in MPFC in patients with major depressive disorder and panic disorder were significantly increased after the treatment [53]. Consistent with these studies, we also found decreased ReHo within MPFC for individuals who have lower level of happiness.

In contrast, higher ReHo was found within left DLPFC for unhappy individuals. One methodological limitation of the ReHo approach is that its biological significance is still unclear, thus it is difficult to interpret the exact meaning of the opposite effects observed within MPFC and DLPFC [42]. However, our findings support the view that these two regions played different functional roles in emotion [54]. One main role of DLPFC is executive control [55], [56]. Happy individuals, relative to unhappy individuals, experienced more positive moods [4], this is possibly due to happy people having greater cognitive control abilities to regulate negative emotional experience [57] and maintain and update positive information in working memory [5]. Evidence from clinical populations further supports the role of DLPFC in emotional regulation. For example, patients with left DLPFC damage have an increasing likelihood of depressive symptoms [52] which is associated with deficits in positive affect [58] In late-life subthreshold depression [22] and major depressive disorder [59] patients, abnormal resting-state ReHo and functional connectivity were also reported within this region.

Besides the DLPFC, the right MCG, right putamen, and left thalamus also exhibited increased ReHo in unhappy individuals. Furthermore, the ReHo score within the left thalamus is negatively correlated with CHI scores in happy group. Previous personality neuroimaging studies demonstrated that the volume of MCG was correlated with neuroticism [60]. Neuroticism is characterized by anxiety, guilt, and emotional instability. Compared with other personality traits, neuroticism is the strongest predictor of happiness [61]. Thus, the increased regional coherence in unhappy individuals within MCG may suggest that unhappy individuals are less emotionally stable and more reactive to stress, compared with their happy peers.

Both the striatum [62]–[64] and the thalamus [65], [66] played a critical role in reward processing. Specifically, the putamen is not only involved in processing reward outcome, but also engaged in anticipating a reward [65], [66] and the thalamus is suggested to be one of the core areas regulating reward processing [62]. Reward is important for driving incentive-based learning, approaching reward object, and inducing positive emotions [67]. Dysfunction in reward processing may lead to mood disorders [68]. Previous studies have reported increased ReHo in right PT in patients with social anxiety disorder [23], [24], and greater functional connectivity between TH and subgenual cingulate in patients with depression [69]. These results support our finding that these two regions were implicated in happiness.

Although the current results provided novel information on our understanding of happiness, several limitations need to be considered. First of all, general trait happiness instead of a specific type of happiness was assessed in the current study. As happiness can be divided into hedonic (pleasure attainment and pain avoidance) and eudaimonic components (meaning and self-realization) [70], refined studies to elucidate the neural underpinning of different types of happiness are needed. Secondly, although we started with a large initial sample, the final sample size is relatively small, which may decrease the statistical power to detect the correlations between ReHo and happiness. Limited power also prevented us from investigating the interaction between happiness and gender on ReHo. As this is still an unexplored question and previous behavioral and neuropsychological studies have reported inconsistent results, it would be of merit to tackle this question in our future study. Finally, recent work showed that ReHo is correlated with neurovascular variables and the contribution of ReHo to task activation can be counted by neurovascular factors [71]. Thus, alterations in ReHo might not purely reflect changes in neuronal activity but instead it might reflect changes in neurovascular coupling, or both. Future work is needed to investigate the physiological significance of ReHo before we can more accurately interpret the direction of the results (e.g. increased or decreased ReHo in certain regions for one group compared with another group).

## Conclusions

In summary, we used a ReHo approach to investigate the differences in intrinsic brain activities between happy and unhappy individuals. We found that the local synchronization of intrinsic brain activities was altered in unhappy individuals within prefrontal cortex, temporal lobe, limbic system, and subcortical regions. These regions overlapped with the previously identified DMN, which suggests that DMN plays an important role in subjective happiness and is in support of the construal theory. Our findings also provide further evidence to support that core components of the emotional and rewarding network are involved in happiness.
